# Supplementary material for: Empirical methods for controlling false positives and estimating confidence in ChIP-Seq peaks
Source: BMC Bioinformatics. 2008 Dec 5;9:523. doi: 10.1186/1471-2105-9-523 (PMC2628906; doi:10.1186/1471-2105-9-523)
Supplement: Additional File 1 — A variety of html documents from the USeq web site detailing the available applications, their best usage, output file type descriptions, command line menus, etc. [file 1471-2105-9-523-S1.zip › USeqUserGuides/JavaDoc.zip!/JavaDoc/trans/tpmap/MapFeature.html]

MapFeature


|  |  |  |
| --- | --- | --- |
| **Overview** \| **Package** \| **Class** \| **Use** \| **Tree** \| **Deprecated** \| **Index** \| **Help** | |  |
| **PREV CLASS**   **NEXT CLASS** | **FRAMES**    **NO FRAMES**     **All Classes** |  |
| SUMMARY: NESTED \| FIELD \| CONSTR \| METHOD | DETAIL: FIELD \| CONSTR \| METHOD |  |


---


## trans.tpmap Class MapFeature

```
java.lang.Object
  trans.tpmap.MapFeature
```

**All Implemented Interfaces:**: java.io.Serializable, java.lang.Comparable

---

``` public class MapFeature extends java.lang.Object implements java.lang.Comparable, java.io.Serializable ```

Tpmap line broken into some components, chromosome, start, pmX, pmY, and optionally, mmX, mmY.
No getters or setters to minimize size and increase spead recall.

**See Also:**: Serialized Form

---

| **Field Summary** | |
| --- | --- |
| `java.lang.String` | `chromosome` |
| `int` | `matches` |
| `int` | `mmX` |
| `int` | `mmY` |
| `int` | `pmX` |
| `int` | `pmY` |
| `static long` | `serialVersionUID` |
| `int` | `start` |


**Constructor Summary**

`MapFeature(java.lang.String tpmapLine)`
  
          Constructor - bpmapLine - seq ori chrom start pmX pmY mmX mmY, (mm coords are optional).


| **Method Summary** | |
| --- | --- |
| `int` | `compareTo(java.lang.Object obj)`             Sorts by chromosome and then position. |

**Methods inherited from class java.lang.Object**

`equals, getClass, hashCode, notify, notifyAll, toString, wait, wait, wait`

**Field Detail**

### serialVersionUID

```
public static final long serialVersionUID
```

**See Also:**: Constant Field Values

---


### chromosome

```
public java.lang.String chromosome
```

---


### matches

```
public int matches
```

---


### start

```
public int start
```

---


### pmX

```
public int pmX
```

---


### pmY

```
public int pmY
```

---


### mmX

```
public int mmX
```

---


### mmY

```
public int mmY
```


**Constructor Detail**

### MapFeature

```
public MapFeature(java.lang.String tpmapLine)
```

:   Constructor - bpmapLine - seq ori chrom start pmX pmY mmX mmY, (mm coords are optional).


**Method Detail**

### compareTo

```
public int compareTo(java.lang.Object obj)
```

:   Sorts by chromosome and then position.

    :   **Specified by:**: `compareTo` in interface `java.lang.Comparable`


---


|  |  |  |
| --- | --- | --- |
| **Overview** \| **Package** \| **Class** \| **Use** \| **Tree** \| **Deprecated** \| **Index** \| **Help** | |  |
| **PREV CLASS**   **NEXT CLASS** | **FRAMES**    **NO FRAMES**     **All Classes** |  |
| SUMMARY: NESTED \| FIELD \| CONSTR \| METHOD | DETAIL: FIELD \| CONSTR \| METHOD |  |


---
